# Dose mapping sensitivity to deformable registration uncertainties in fractionated radiotherapy – applied to prostate proton treatments

**DOI:** 10.1186/1756-6649-13-2

**Published:** 2013-06-14

**Authors:** David Tilly, Nina Tilly, Anders Ahnesjö

**Affiliations:** 1 Department of Radiology, Oncology and Radiation Sciences, Uppsala University, Uppsala, Sweden; 2 Elekta Instruments AB, Uppsala, 753 21, Sweden

**Keywords:** Radiotherapy, Adaptive radiotherapy, Dose tracking, Dose mapping, Dose accumulation, Dose accumulation accuracy, Deformable image registration, Non-rigid image registration, Protons

## Abstract

**Background:**

Calculation of accumulated dose in fractionated radiotherapy based on spatial mapping of the dose points generally requires deformable image registration (DIR). The accuracy of the accumulated dose thus depends heavily on the DIR quality. This motivates investigations of how the registration uncertainty influences dose planning objectives and treatment outcome predictions.

A framework was developed where the dose mapping can be associated with a variable known uncertainty to simulate the DIR uncertainties in a clinical workflow. The framework enabled us to study the dependence of dose planning metrics, and the predicted treatment outcome, on the DIR uncertainty. The additional planning margin needed to compensate for the dose mapping uncertainties can also be determined. We applied the simulation framework to a hypofractionated proton treatment of the prostate using two different scanning beam spot sizes to also study the dose mapping sensitivity to penumbra widths.

**Results:**

The planning parameter most sensitive to the DIR uncertainty was found to be the target *D*_95_. We found that the registration mean absolute error needs to be ≤0.20 cm to obtain an uncertainty better than 3% of the calculated *D*_95_ for intermediate sized penumbras. Use of larger margins in constructing PTV from CTV relaxed the registration uncertainty requirements to the cost of increased dose burdens to the surrounding organs at risk.

**Conclusions:**

The DIR uncertainty requirements should be considered in an adaptive radiotherapy workflow since this uncertainty can have significant impact on the accumulated dose. The simulation framework enabled quantification of the accuracy requirement for DIR algorithms to provide satisfactory clinical accuracy in the accumulated dose.

## Background

The patient geometry can change significantly between fractions during radiotherapy treatments. This motivates the use of image sets to monitor the treatment progress and to serve as basis for optional re-planning to better fulfil the treatment objectives.

The ideal monitoring scenario would be to score dose for each individual tissue part, or cell, throughout all delivered treatment fractions. The closest realisation of such a scenario is to calculate dose on images taken for each treatment occasion, and use deformable image registration (DIR) to map and accumulate the dose contributions onto a representative, single image dataset [1]. Such dose distributions can be compared with the intended, planned dose distribution to provide a basis for corrective interventions in adaptive radiotherapy. The accuracy of the image registration will govern the accuracy of the mapped dose, and hence the relevance of the corrections. The validity of the accumulated dose, due to the image registration uncertainty, was recently debated by Schultheiss and Tomé [2]. It is therefore of fundamental interest to determine the accuracy requirements of the image registration process, separate from other uncertainties, in particular with regards to the accuracy of the quantities derived from the finally mapped dose.

The input to the registration also contains uncertainties, e.g. image noise and uncertain segmentations. The registration uncertainty can be considered as uncorrelated to other treatment uncertainties such as organ motion effects or dose calculation uncertainties, and can thus be investigated separately. There exists a plethora of DIR algorithms in the literature, see e.g. [3,4] for a review of available methods. Studies of the accuracy of different DIR algorithms applied to different body sites exist [5,6] with reported average errors in the range of 1–5 mm. The accuracy of dose mapping has also been studied explicitly by several authors; Rosu *et al.*[7] investigated dose grid resolution effects, Salguero *et al.*[8] utilised the image registration inverse inconsistency and found a maximum dose mapping uncertainty of >30% of the prescription dose for a lung patient, Yan *et al.*[9] related the lack of mass conservation in the registration to the dose uncertainty, Hub *et al.*[10] estimated the dose uncertainty from the registration parameter uncertainty and Murphy *et al.*[11] developed a method to sample image registration errors and demonstrated their effect on the mapped dose.

The aim of this study is to develop a methodology for determining the accuracy requirements for the use of deformable image registration as a basis for dose accumulation in fractionated radiotherapy. For this purpose we developed a simulation framework for dose accumulation where the uncertainty of the calculated treatment outcome (represented as dose, or as dose response determined with a biological models) as a function of the registration uncertainty could be investigated. The dosimetric impact of the registration uncertainty on the entire fractionated treatment has been studied by Risholm *et al.*[12] who presented the uncertainty of the total physical dose for a single estimated registration uncertainty, while we study how the final dose and the related response would vary with the registration uncertainty.

We apply the framework to a hypofractionated treatment of the prostate with spot scanned protons where a “plan of the day” is assumed to be tailored to the CTV for each individual fraction. The clinical importance of dose accumulation for prostate treatments [13] was investigated in a recent publication by Wen *et al.* We hypothesise that the sensitivity to dose mapping uncertainty increases with steeper dose gradients and therefore we use dose distributions from two different spot sizes, one based on measurements at the local clinic and one with smaller spot sizes producing a sharper and less forgiving penumbra. We also investigate the accuracy requirement versus the required size of the planning margin used for construction of the planning target volume (PTV) from the clinical target volume (CTV).

## Methods

The impact of the registration uncertainty on the accumulated dose, and its estimated response, can be studied by adding known uncertainties to the registrations used in mapping the deformations. The simulations mimic a clinical workflow where a “plan of the day” is tailored to the actual position of the CTV for the individual fractions. The total accumulated effect, i.e. the estimated response for the dose mapped from all fractions to a fixed reference geometry is scored for various degree of registration uncertainties. As we are specifically interested in the effects of the registration uncertainty we assume that the patient is imaged and setup without errors at each fraction. According to the van Herk margin scheme [14] this means that we simulate a situation yielding only the random contribution from DIR based dose mapping to the CTV to PTV margin. In a real clinical situation also other sources of uncertainty must be considered. In section A we describe the simulation framework and the modelling of the image registration uncertainty, and in section B we apply the uncertainty model to a hypofractionated prostate treatment with spot scanned protons.

### A. Simulation of a fractionated treatment with dose mapping uncertainties

**Figure 1:**
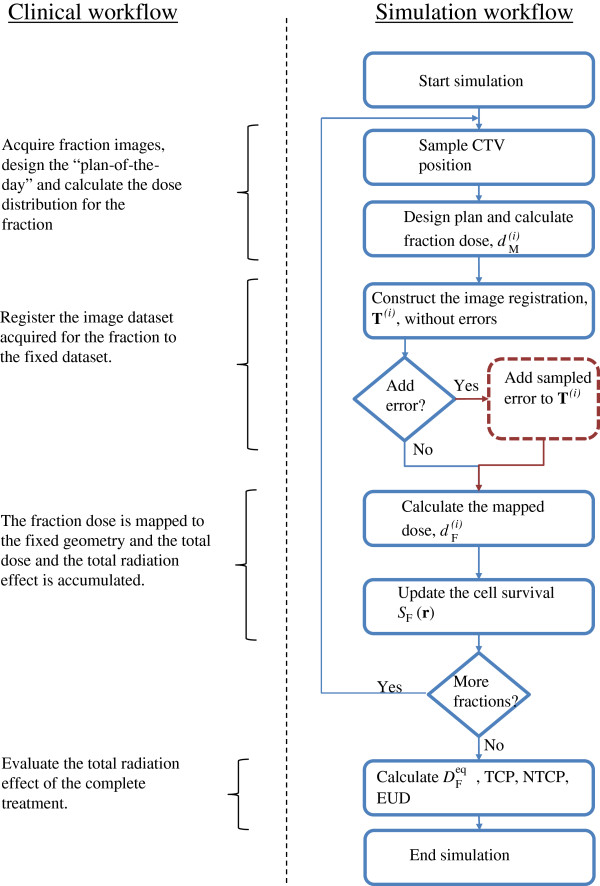
**Simulated clinical workflow.** The flow chart to the right shows how the clinical workflow, listed to the left, is simulated. The fraction dose is calculated on a randomly deformed virtual patient model for which the exact image registration to the fixed geometry *I*_F_ is known. A registration uncertainty can be added (the red part of the flow chart), before the dose is mapped to *I*_F_ where the radiation effect is accumulated.

A “plan of the day” is prepared for each of the *N* treatment fractions, and the fraction dose is mapped to a reference (fixed) image set *I*_F_ for evaluation of the cumulative radiation effect, see Figure 1. For each fraction *i* a moving image set $I_{M}^{\left(i\right)}$ is acquired, and the delivered fraction dose $d_{M}^{\left(i\right)}$ for fraction *i* is assumed to be calculated based on $I_{M}^{\left(i\right)}$ It is further assumed that through the use of DIR, the transformation **T**^(*i*)^(**r**) that best align $I_{M}^{\left(i\right)}$ with *I*_F_ can be determined so that each point **r** in *I*_F_ is mapped to a corresponding point in $I_{M}^{\left(i\right)}$ by **T**^(*i*)^ and by means of interpolation in $d_{M}^{\left(i\right)}$, the dose is mapped from $I_{M}^{\left(i\right)}$ to *I*_F_ through

(1) $$d_{F}^{\left(i\right)}\left(r\right)=d_{M}^{\left(i\right)}\left(T^{\left(i\right)}\left(r\right)\right)$$

to enable estimation of accumulated dose for all fractions through

(2) $$D_{F}\left(r\right)=\sum_{i=1}^{N}d_{F}^{\left(i\right)}\left(r\right)=\sum_{i=1}^{N}d_{M}^{\left(i\right)}\left(T^{\left(i\right)}\left(r\right)\right).$$

The dose distribution $d_{F}^{\left(i\right)}\left(r\right)$ will vary from fraction to fraction because of organ motion and the “plan of the day” approach. We adopt a view where we with a given uncertainty can track the position of a tissue element at all treatments and accumulate the radiobiological effect fraction by fraction. The *D*_F_(**r**) then becomes a stochastic quantity.

The resulting radiobiological effect was modelled based on the LQ-model [15]. The 4D dose distribution is thereby converted into a 3D equivalent fraction dose $d_{F}^{\mathrm{eq}}\left(r\right)$[16], calculated as the fraction dose giving the equivalent total radiobiological effect, *S*_F_(**r**), by solving for $d_{F}^{\mathrm{eq}}\left(r\right)$ from

(3) $$\begin{array}{ll} \;S_{F}\left(r\right) & =\prod_{i=1}^{N}\exp\left(-\alpha d_{F}^{\left(i\right)}\left(r\right)-\beta d_{F}^{\left(i\right)}{\left(r\right)}^{2}\right)\\ & ={\left[\exp\left(-\alpha d_{F}^{\mathrm{eq}}\left(r\right)-\beta d_{F}^{\mathrm{eq}}{\left(r\right)}^{2}\right)\right]}^{N}. \end{array}$$

The total equivalent dose, $D_{F}^{\mathrm{eq}}\left(r\right)=Nd_{F}^{\mathrm{eq}}\left(r\right)$ was used for analysis of the treatment DVHs. The tumour control probability, TCP, was calculated directly from *S*_F_(**r**), c.f. [17,18]. The normal tissue complication probability, NTCP, was calculated using the Lyman model [19] with parameters from Burman *et al.*[20] and Emami *et al.*[21]. Also the equivalent uniform dose, EUD [22], was calculated from $D_{F}^{\mathrm{eq}}$.

#### Modelling the image registration uncertainty

The transformation **T**^(*i*)^(**r**) between the fixed image *I*_F_ and the moving image $I_{M}^{\left(i\right)}$, used by the dose mapping procedure in equation (1), was modelled through

(4) $$T^{\left(i\right)}\left(r\right)=T_{0}^{\left(i\right)}\left(r\right)+T_{e}^{\left(i\right)}\left(r\right)$$

where $T_{0}^{\left(i\right)}$ is the true transformation, and the transformation error $T_{e}^{\left(i\right)}$ models the registration uncertainty. Neither $T_{0}^{\left(i\right)}$ or $T_{e}^{\left(i\right)}$ are in general known for a clinical situation and thus **T**^(*i*)^ have to be treated as a stochastic quantity for which we assume that $\langle T_{e}^{\left(i\right)}\left(r\right)\rangle =0.$ Registration algorithms apply various regularisation techniques to produce a well behaved and physically realistic transformation. Clearly, there is a local correlation of the error for points close together whereas for points further apart the error will be more uncorrelated. In modelling of $T_{e}^{\left(i\right)}$ this is mimicked by sampling uncorrelated errors at a sparse grid of control points which by means of a 3D cubic B-Spline interpolation is applied to the denser dose grid. The registration deviation amplitudes is sampled at the control points, independently for each coordinate according to a multinormal distribution with zero mean and standard deviation *σ*_e_. A low resolution of the control points will produce a slowly varying vector field and thus mimic a high degree of regularisation and vice versa for a high resolution of the control points.

Registration algorithms are often validated using landmarks where the distances between known displacements of the landmarks and those calculated by the algorithm, which in our case is equivalent to the absolute registration error $\left|T_{e}^{\left(i\right)}\left(r\right)\right|,$ are compared. The relationship between the standard deviation of the control point distribution, *σ*_e_, and the mean absolute registration error, $\langle \left|T_{e}^{\left(i\right)}\left(r\right)\right|\rangle ,$ can be calculated using the B-Spline interpolation coefficients (independent of the B-Spline resolution) and is found to be

(5) $$\langle \left|T_{e}\right|\rangle \approx 0.534\;\sigma_{e}$$

### B. Application of the simulation framework to a virtual prostate patient

#### The virtual patient model

**Figure 2:**
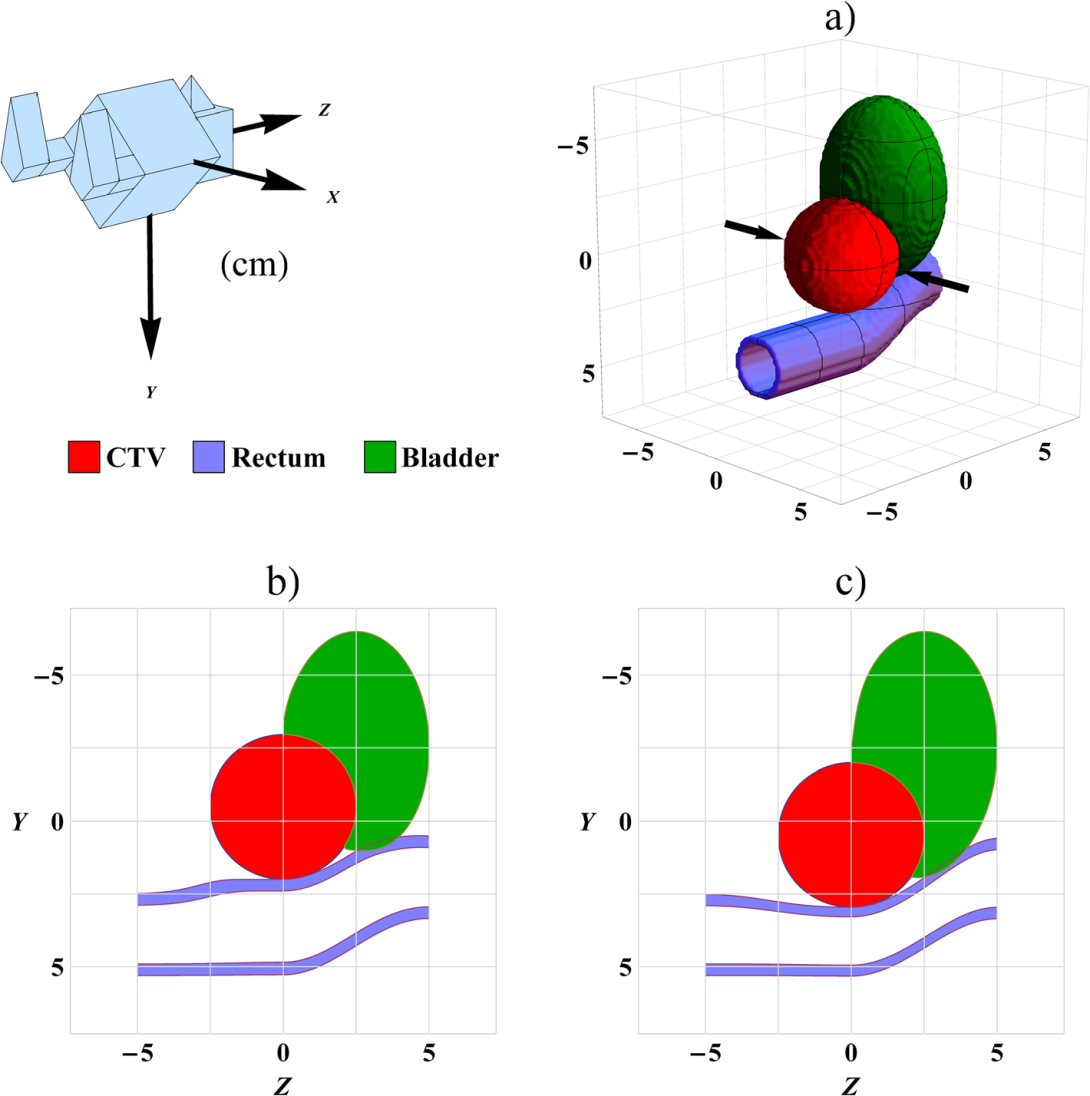
**The virtual patient and its deformation.** The patient position and the coordinate system are indicated in the top left corner. (**a**) 3D view of the virtual patient with the centre of the CTV at the origin. The black arrows indicate the direction of the two incident scanned proton beams. (**b**) A slice at x = 0.0 cm of the virtual patient in which the prostate CTV has moved 0.5 cm versus the reference in (**a**) in the negative y-direction (Anterior-Posterior) causing an intrusion into the bladder and an expansion of the rectum. (**c**) The CTV has moved 0.5 cm in the positive y-direction compressing the rectum and decreasing the bladder intrusion.

The framework described in section A require a patient geometry, $I_{M}^{\left(i\right)}$, and its deformation for every fraction $T_{0}^{\left(i\right)}$. A single patient instance is defined as a patient with a unique geometry per fraction. In the simulation we used ten different patient instances for evaluation of each parameter combination. A male pelvic virtual patient was for this purpose constructed from average data for 15 prostate patients treated in the supine position, see Figure 2. The prostate was modelled as a sphere with radius *r*_CTV_ = 2.5 cm located at 20 cm depth and the bladder as an ellipsoid with radii 3.0 cm, 4.0 cm and 2.5 cm in the lateral, superior-inferior and axial directions, respectively, with a concave intrusion from the presence of the prostate. The rectum was modelled by a curved cylinder with outer radius 1.4 cm and length 10.0 cm with wall thickness 0.4 cm [23]. Other anatomical details, such as the femoral heads, were not included since they are in principle uncorrelated with the image registration dose mapping effects for the prostate, bladder and rectum regions.

The prostate is assumed to be incompressible and change location (but not shape) as the rectum and bladder change filling and shape. In prostate radiotherapy treatment it is common to also irradiate the lower parts of the seminal vesicles to the same dose as the prostate. The combined volume, prostate and lower parts of the vesicles, will constitute a rather convex volume which we for simplicity model as a sphere shaped CTV. We sample the displacement of the prostate centre for each fraction, $v_{\mathrm{CTV}}^{\left(i\right)},$ according to a multinormal distribution with the standard deviations 0.4, 0.1 and 0.4 cm in the anterior-posterior, lateral and superior-inferior directions, respectively, consistent with the literature [24]. The fixed geometry *I*_F_, into which the radiation effect is accumulated, is simply chosen to be the one with zero displacement of the prostate, cf. Figure 2a.

**Figure 3:**
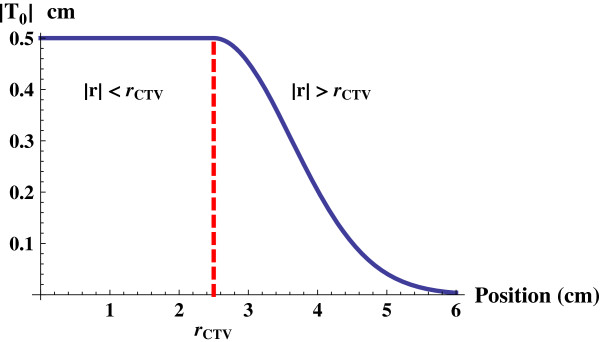
**Tissue displacement model.** The absolute value of the tissue displacement, |**T**_0_|, as a function of the distance |**r**| from the prostate centre, when a prostate with radius 2.5 cm is assumed to be displaced 0.5 cm. Inside the prostate, i.e. |**r**| < *r*_CTV_, the displacement is constant. Outside the CTV, i.e. |**r**| > *r*_CTV_, the displacement decreases exponentially and vanishes far from the prostate

A simple tissue deformation model was used to construct the true moving geometry $I_{M}^{\left(i\right)}$ from *I*_F_ where the tissue displacement outside the prostate in *I*_F_ is exponentially relaxed with the squared distance from the prostate edge according to

(6) $$T_{0}^{\left(i\right)}\left(v_{\mathrm{CTV}}^{\left(i\right)},r\right)=v_{\mathrm{CTV}}^{\left(i\right)}.\exp\left(-k\cdot \frac{{\left(\left|r\right|-r_{\mathrm{CTV}}\right)}^{2}}{{\left|v_{\mathrm{CTV}}\right|}^{2}}\right)\;\left|r\right|>r_{\mathrm{CTV}},$$

where **T**_0_ is the local displacement at position **r**. The relaxation parameter *k* was set to 0.1 to give reasonable volume differences in the rectum. The tissue displacement according to equation (6) for a prostate displacement $\left|v_{\mathrm{CTV}}^{\left(i\right)}\right|\;$ = 0.5 cm and *k* = 0.1 is shown in Figure 3. The virtual patient is shown for two prostate displacements in Figure 2b and c where the rectum and bladder are deformed according to the deformation model in equation (6). The model does not assume anything about the reasons why the prostate has moved, i.e. the motion can be seen as a consequence of the filling in the rectum or the bladder.

The dose grid resolution was set to 0.1 cm while two different grid sizes, 1.0 cm and 3.0 cm, was used for the control points in the registration error. Analysis of all **T**^(*i*)^(**r**), i.e. the patient model and the registration error, showed values of the Jacobian determinant, *J*(**T**^(*i*)^(**r**), **r**), in the interval [0.4, 2.0] for the lower resolution indicating an inverse consistent transform without folds or tears since *J* > 0. The **T**^(*i*)^(**r**) produced using the higher control point resolution included small regions with negative values of the *J* for σ_e_ ≥ 0.5 cm indicating a transform that is not well regularised. It is desirable that the accumulated dose is independent of the choice of reference image *I*_F_ and this requires that *J* > 0, which is not in general guaranteed by registration algorithms. The resulting dose mapping uncertainty was very similar for both resolutions but slightly more sensitive with respect to the image registration uncertainty for the 3.0 cm resolution. We have therefore concentrated on results for the 3.0 cm resolution.

### Generation of treatment plans for simulation

The simulated treatments were assumed to be delivered with two opposed, scanned proton beams, indicated by the arrows in Figure 2a, aiming for a homogenous target dose.

The PTV was constructed by adding a margin *m* isotropically around the CTV.

Clinically relevant planning objectives were chosen from literature values for intermediate risk patients for radiobiological evaluation of the prostate [25], see Table 1, to optimise the spot weights and thus shape the proton dose distribution. Additionally, an artificial OAR was created as a spherical shell around the PTV to suppress the normal tissue dose. The target dose prescription was chosen to give a TCP of 80% since the TCP curve is steep at that dose level and thus the TCP should be susceptible to dose mapping uncertainties. However, because of the suggested low *α*/*β* there is a trend towards applying hypofractionated treatments, see e.g. Ritter [26]. Therefore, an aggressive hypofractionation scheme of 6.7 Gy × 5fx still aiming for a TCP of 80% were chosen with the OAR planning objectives from Isacsson *et al.*[27] scaled with the new prescription dose. Zavgorodni [28] noted that it might be important to take the variable fraction dose into account, as in equation (3), when accumulating the dose for normal tissue and tumours with low *α*/*β*, such as the prostate, and for hypofractionated treatments.

**Table 1:** Summary of the treatment planning objectives and the radiobiological parameters used in the evaluation

**ROI**		***α*/*β***
CTV	N/A	1.5
PTV	Minimise *V*(*D*<33.0 Gy)	N/A
	Minimise *V*(*D*>34.7 Gy)	
Rectum	Minimise *V*(*D*>29.6 Gy)	3.0
Bladder	Minimise *V*(*D*>29.6 Gy)	3.0

The “plan of the day” treatment scenario described above requires a tailored dose distribution for each target position for all patient instances, i.e. $d_{M}^{\left(i\right)}$ in equation (1). To save computation time and make the result less dependent of the intrinsic details of the optimiser we displace the dose distribution according to the fraction specific CTV displacement. This results in one optimisation per margin size and spot size regimen.

The scanned proton dose distribution was calculated using an in-house pencil beam algorithm with the depth dose and lateral scattering calculated according to Bortfeld [29] and Russell *et al.*[30], respectively. Our normal beam model (NBM) parameters are based on data from Kimstrand *et al.*[31]. Dose mapping in the presence of a sharp penumbra will be sensitive to the registration uncertainty, especially for targets where the loss of dose coverage can greatly affect the outcome. Dose distributions were therefore also generated according to a sharp penumbra beam model (SBM) whose in-air spot sizes are smaller and with zero divergence thus producing a sharper penumbra. The spot sizes of SBM correspond approximately to what modern commercial proton machines can deliver, e.g. see [32]. The penumbra width is often reported as the distance that the dose falls from 80% to 20% of the target dose and here they were 1.16 and 0.88 cm for NBM and SBM respectively in the directions perpendicular to the beam axis. The spot size increase with treatment depth, due to multiple scatter of the protons, and note that a shallower target will have a sharper penumbra and thus more sensitive to dose mapping uncertainties.

## Results

The simulations were performed for ten different instances of the virtual patient where each instant had its own set of fraction specific CTV positions with corresponding deformation fields. A single treatment simulation, according to Figure 1, modelled the treatment of a patient instance with the prescribed 6.7 Gy × 5fx. To ensure adequate statistics, each treatment simulation for the same patient instance was repeated 40 times with different sampled image registration **T**^(*i*)^ according to equation (5) for each value of the margin *m* (0.0, 0.1, …, 0.4 cm), image registration uncertainty 〈|**T**_e_|〉  (0.0, 0.05, …,0.4 cm) and beam models. This resulted in a total of 400 treatment simulations per combination of *m*, 〈|**T**_e_|〉 and beam model.

### The DVH dependency on varying registration uncertainty

**Figure 4:**
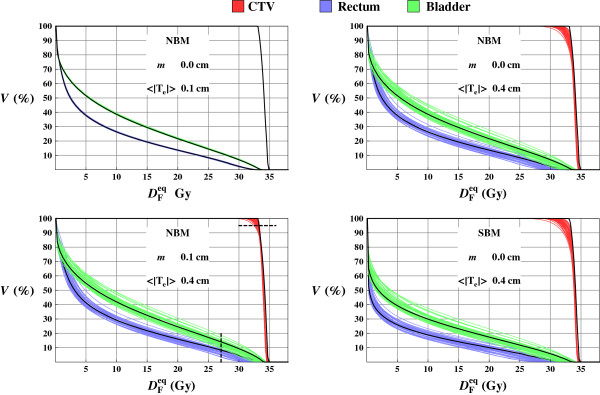
**DVHs from 5fx treatments simulations.** DVHs resulting from 5fx treatment simulations for a single patient instance where each individual plot contains 40 different treatment simulations using the same margin *m*, and beam model but different image registration uncertainty distributions (sampled with the same 〈|**T**_e_|〉). The nominal DVHs without dose mapping uncertainty is drawn in black as a reference. The *D*_95_ and *V*_80_ levels, used in the analysis, are indicated with dashed lines in lower left panel.

The spread of the calculated DVH curves for different registration uncertainties is shown in Figure 4 including the nominal DVH with no registration uncertainty as a reference. As expected, the spread of the DVH curves increases when the registration uncertainty 〈|**T**_e_|〉 increases, c.f. the upper left panel with the rest in Figure 4. For the CTV, use of a planning margin (*m* > 0 cm) reduces the sensitivity to the registration uncertainty, and the spread of the DVH curves decreases compared to planning without margin (*m* = 0 cm). Hence, an increased target margin reduces the difference in dose between the target and surrounding normal tissue and therefore reduces the effect of the registration uncertainty. A sharper penumbra, i.e. created by the SBM instead of the NBM, gives a slightly increased sensitivity to registration uncertainty.

The spread of the DVHs for the OAR is less dependent of the planning margin and the penumbra width, although the overall dose level increases when any of these two parameters increases. The dose tend to be overestimated in the low dose region but underestimated in high dose regions which is clearly seen for the rectum and bladder in Figure 4. The reason for the underestimation for the high dose is similar as for the target dose, a dose mapping uncertainty is more likely to lower the dose (and vice versa for the low dose region).

**Figure 5:**
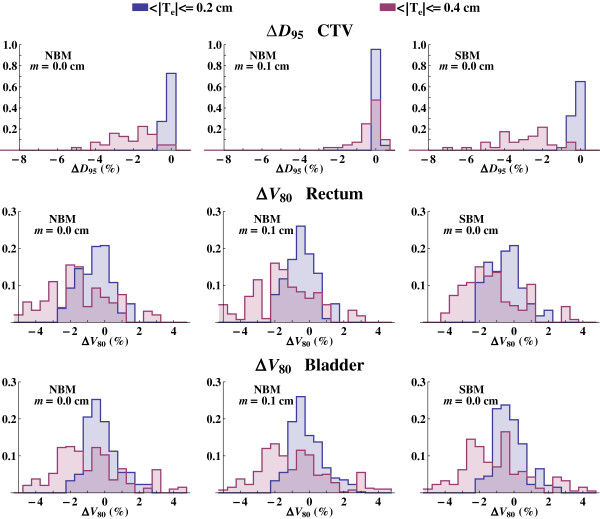
**The *D***_**95 **_**and *V***_**80 **_**distributions for different image registration uncertainties.** The frequency distributions of the uncertainty in *D*_95_ (CTV) and *V*_80_ (OARs), i.e. ∆*D*_95_ and ∆*V*_80_, due to dose mapping uncertainty, for all ten instances of the patient, as a result of the image registration uncertainties 〈|**T**_*e*_|〉 equal to 0.2 and 0.4 cm. The leftmost column shows the distribution for dose distributions using the NBM and no planning margin, the middle column using a planning margin *m* and the right column the result for the dose distribution using the SBM.

The dose spread for the 95% of the CTV volume, *D*_95_, and the volume of the OARs that receives 80% of the prescription dose, *V*_80_, is chosen to further illustrate the spread of the DVHs. The frequency distributions of ∆*D*_95_ and ∆*V*_80_ expressed as the change in *D*_95_ and *V*_80_ versus the nominal values are shown in Figure 5 for all sampled patient instances. These results demonstrate that the registration uncertainty causes a decreased value of the calculated *D*_95_ (∆*D*_95_ ≤ 0%) for the target. It is evident that an increased planning margin will decrease the impact of the dose mapping uncertainty. There is also a trend that *V*_80_ gets underestimated as the dose mapping uncertainty increases.

**Figure 6:**
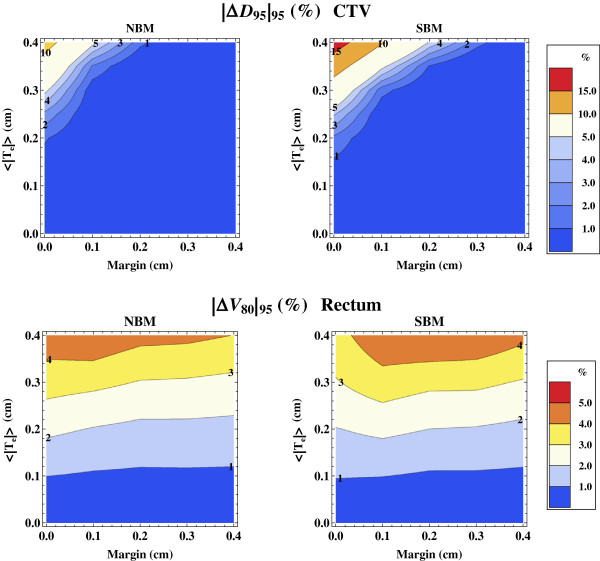
**The relative difference in *D***_**95 **_**and *V***_**80 **_**encompassing 95% of the treatment simulations.** (Upper) Isolevels of the relative dose difference versus the nominal DVH (no dose mapping uncertainty, i.e. 〈|**T**_e_|〉 = 0 cm) that include 95% of all treatment simulation outcomes, |*ΔD*_95_|_95_, as a function of the image registration uncertainty, 〈|**T**_e_|〉, and planning margin shown for the two beam models NBM and SBM. (Lower) Isolevels of the distance in relative volume at 80% of the target dose to the nominal DVH that include 95% of all outcome, |*ΔV*_80_|_95_, for the rectum.

The absolute values of ∆*D*_95_ and ∆*V*_80_ that include 95% of all the DVH curves, i.e. |Δ*D*_95_|_95_ and |Δ*V*_80_|_95_ are shown in Figure 6 for dose distributions from beam models NMB and SBM. As expected, the |Δ*D*_95_|_95_ is decreased when the planning margin is increased, or when a wider penumbra (NBM) is used.

To obtain the DIR accuracy such that |Δ*D*_95_|_95_ is approximately 3% of the target dose, we found that the registration uncertainty 〈|**T**_e_|〉 must be less than 0.25 cm for NBM and SBM with SBM slightly more sensitive to 〈|**T**_e_|〉. If a planning margin of 0.1 cm is used, the 〈|**T**_e_|〉 registration uncertainty can be relaxed to 0.35 cm for NBM and 0.30 cm for SBM.

The value of |Δ*V*_80_|_95_ was found to increase with increasing 〈|**T**_e_|〉 and weakly with increasing margin, as shown in the lower plots in Figure 6. The |Δ*V*_80_|_95_ value was found to increase linearly as a function of 〈|**T**_e_|〉, and was approximately 1% and 4% of the ROI volume for 〈|**T**_e_|〉 = 0.1 cm and 0.35 cm, respectively. The |Δ*V*_80_|_95_ increases weakly with increased margin and the difference between NBM and SBM is small. The results for the bladder |Δ*V*_80_|_95_ were similar as the data found for the rectum.

### The uncertainty in simulated radiation response

The sensitivity of the calculated radiobiological outcome quantities TCP and EUD for the CTV, and NTCP and EUD for the OARs, were determined for each treatment simulation. The chosen technique yields very low nominal NTCP values, less than 2% for the rectum and much smaller for the bladder. Accordingly, the EUD uncertainties for the risk organs were also very small, < 0.5 Gy.

**Figure 7:**
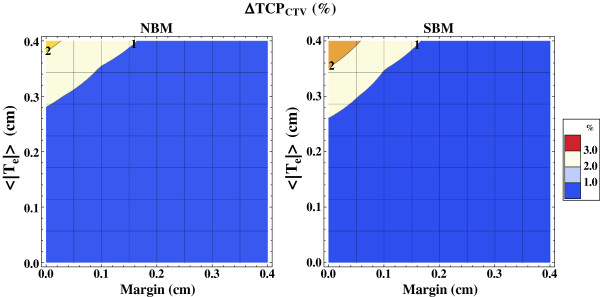
**The uncertainty in TCP due to dose mapping errors.** Isolevels of the decrease in the calculated TCP for the CTV, ∆TCP_CTV_, due to dose mapping errors caused by the image registration uncertainty 〈|**T**_e_|〉, shown for different planning margins *m*, and beam models NBM (left) and SBM (right).

The decrease in TCP versus the nominal without dose mapping uncertainty, ∆TCP_CTV_, was found to increase with increasing registration uncertainty as shown in Figure 7. These results are analogous to the results of the DVH analysis in that the sensitivity to the registration uncertainty is reduced with increasing planning margin, and/or a shallower penumbra. The ∆TCP_CTV_ was below 1% for both beam models NBM and SBM as long as the registration uncertainty 〈|**T**_e_|〉 was less than 0.25 cm. The standard deviation of the ∆TCP_CTV_ (not shown) was small (<1%). A similar analysis of the ∆EUD_CTV_ yielded quantitatively very similar results as for the ∆TCP_CTV_.

### The distribution of spatial dosimetric uncertainty with varying registration uncertainty

**Figure 8:**
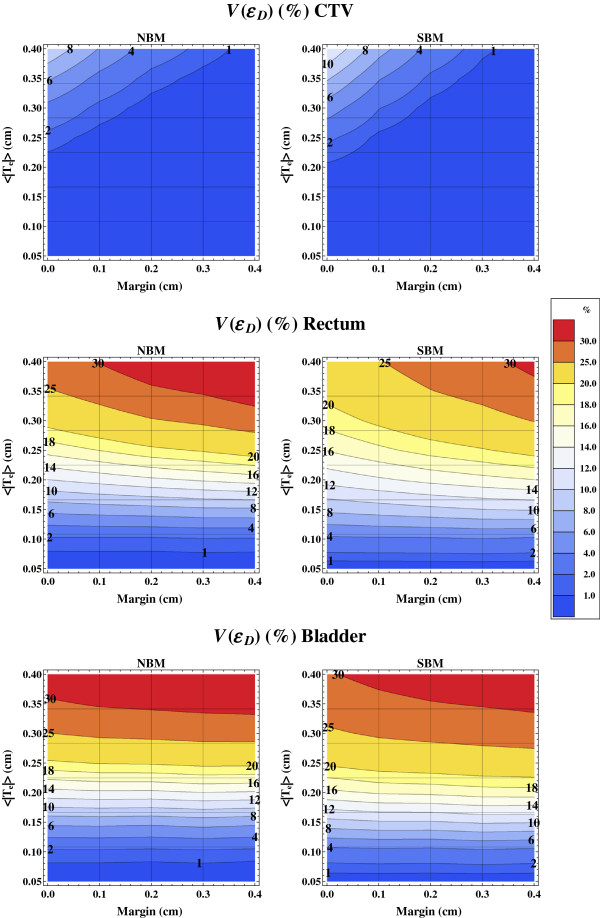
**Summed relative volume with at least 3% dose mapping uncertainty.** Isolevels of the summed relative volume *V*(*ϵ*_*D*_) that has a dose mapping uncertainty of more than *ϵ*_*D*_ = 3% (relative to the target prescription dose) as a function of the planning margin and registration uncertainty 〈|**T**_e_|〉 shown for the CTV (upper), rectum (middle), bladder (lower) and beam models NBM (left) and SBM (right).

In order to analyse the spatial effects of dose mapping uncertainties we investigated how large volumes, *V*(*ϵ*_*D*_) that got local dose mapping uncertainty (relative to the target prescription dose) larger than *ϵ*_*D*_. The *V*(3%) is shown in Figure 8 for the CTV, rectum and bladder for the beam models NBM and SBM. Without a planning margin (*m* = 0.0 cm), the *V*(3%) for the CTV vary between <1% and up to 10% as the 〈|**T**_e_|〉 vary between 0.2 cm and 0.4 cm. If a margin is applied the *V*(3%) decreases. The *V*(3%) vary in a logarithmic fashion for both rectum and bladder with the registration uncertainty. The use of a planning margin, or the larger spot sizes of NBM, result in larger *V*(3%) for the OARs, because of increased dose burden, but with a similar logarithmic behaviour with the registration uncertainty.

## Discussion

We have in this work developed a general framework for studying the influence image registration uncertainties have on the accuracy of the accumulated dose in fractionated radiotherapy. This framework was used to estimate the impact of the dose mapping uncertainties on the radiobiological quantities such as the TCP as well as the DVH quantities *D*_95_ and *V*_80_ for spot scanned proton therapy of the prostate. The results should be considered together to yield a complete view of the registration accuracy requirements. The relation between the TCP uncertainty and the DVH dose uncertainty can be illustrated by assuming a homogenous CTV dose where a 1% decrease would yield a 2.5% drop in the TCP. The decrease in *D*_95_ is not the same as a decreased homogenous dose but it seems reasonable to require at least a 3% accuracy in *D*_95_ and *V*_80_. A |Δ*D*_95_|_95_  of <3% would require that the mean absolute registration error should be better than approximately 0.25 and 0.20 cm for the larger and smaller spot size respectively, whereas a ∆TCP requirement of <2% would require a mean registration uncertainty better than 0.35 cm. An added planning margin of 0.1 cm significantly reduces the accuracy requirement to 0.35 cm and 0.30 cm for the same *D*_95_ requirement for the larger and smaller spot size respectively. A |Δ*D*_95_|_95_ <2% requirement yields a registration uncertainty requirement of 0.2 cm for the smaller spot size. A requirement of |Δ*V*_80_|_95_  <3% would require a registration uncertainty of 0.25-0.30 cm regardless of spot size and margin. If the 3% requirement on *D*_95_ is fulfilled for the intermediate penumbra, the summed volume with dose mapping uncertainty of more than 3%, in relative dose, *V*(3%), is small for the CTV but up to 15-20% for the organ at risks. The results indicate that the limiting accuracy requirement is that for the *D*_95_. A relaxed penumbra, or an increased target margin, decreases the uncertainty in the CTV while it, due to the increased dose burden, increases the uncertainties in the organs at risk. Jaffray *et al.*[33] highlighted the need for accurate dose accumulation for normal tissue and in that case the large *V*(3%) might be of concern, especially when accumulating dose to serial organs sensitive to local dose effects.

The result of our particular simulations would to some degree depend on the choices of treatment site, patient geometry and deformation method. A shallower target would sharpen the penumbra of the proton treatment and increase the registration accuracy requirement. We assume a registration algorithm without systematic errors and stochastic, uncorrelated random deviations with a spatial frequency resulting from regularisation such that the registration error could be represented as a B-spline vector field with normal distributed control points. We consider these conditions for the registration uncertainty to be rather general for any regularised algorithm with random errors, although its applicability for certain clinically used DIR routines must be checked explicitly. The dosimetric parameters are scored locally ROI by ROI making the conclusions less sensitive to the fact that the DIR uncertainty in reality is not evenly distributed in space. Studies show [5,6,34] that registration algorithms can in some cases, e.g. lung and liver CT-CT registration, have a mean absolute error close to 0.1 cm and thus fulfilling the 3% above stated requirement for *D*_95_.

The uncertainty in the calculation of TCP obviously depends on the slope of the TCP sigmoid shape response curve, i.e. the *γ*_50_ value. The parameters in this work were taken from the analysis of Cheung *et al.*[25] where *γ*_50_ = 2.2 (95% CI 1.1, 3.2). A higher *γ*_50_ would increase the sensitivity to an image registration uncertainty and thus increase ∆TCP. Roughly, using *γ*_50_ = 3.2 instead of 2.2 would raise ∆TCP from 3% to approximately 4.4%, assuming the equivalent relative drop in dose when starting from TCP = 80%.

Future research using the presented framework could include other deformation models and other delivery techniques such as rotational therapy with photons.

## Conclusions

We developed a simulation framework that described the total accumulated dose and the related response as a function of the deformable image registration uncertainty.

When applied to a spot scanned prostate treatment, the accuracy of *D*_95_ on the accumulated dose is a limiting requirement on the deformable image registration when performing dose mapping. A required accuracy of 3% in *D*_95_ would require a mean absolute image registration error uncertainty of 0.20 cm. By increasing the CTV to PTV planning margin with 0.1 cm, the mean absolute error can be relaxed to 0.3 cm with respect to the *D*_95_ requirement. A few algorithms have reported accuracy better than 0.2 cm uncertainty for CT-CT registrations and thus meet the above stated [5,6,34]. However, for more challenging registration problems, such as multi-modality registrations, more development might be required to improve the registration accuracy, or to establish its accuracy as to define the dose mapping contribution to margin requirements in adaptive radiotherapy.

## Competing interests

All authors are, or has been, employed by Elekta Instruments AB.

## Authors’ contributions

DTY and AA conceived and designed the study. DTY performed all software development, executed simulations, data analysis and drafted the manuscript. All authors participated in the simulation design and data interpretation. All authors took part in writing the manuscript and have approved the final manuscript.

## Pre-publication history

The pre-publication history for this paper can be accessed here:

http://www.biomedcentral.com/1756-6649/13/2/prepub
